# Mitochondrial DNA Mutations and Rheumatic Heart Diseases

**DOI:** 10.3390/jcdd6040036

**Published:** 2019-10-11

**Authors:** Fatou Balla Wade, Marie Parsine Sall, Fatimata Mbaye, Mbacké Sembene

**Affiliations:** Genetics and Population Management Team, Department of Animal Biology, Faculty of Sciences and Techniques, Cheikh Anta Diop University, Dakar 5005, Senegal; mparsine@yahoo.fr (M.P.S.); fatimata.mbaye@ucad.edu.sn (F.M.); mbacke.sembene@ucad.edu.sn (M.S.)

**Keywords:** acute rheumatic fever, rheumatic heart diseases, *MT-CYB*, mutation, Senegal

## Abstract

Acute rheumatic fever (ARF) is an autoimmune disease affecting the heart-valve endocardium in its final stage. Although rare in developing countries, ARF persists in third-world countries, particularly Senegal, where rheumatic heart diseases (RHDs) are the most common pediatric cardiovascular pathology. This study aimed to investigate mutations in *MT-CYB* in ARF and RHD in Senegalese patients. *MT-CYB* was amplified from blood samples from ARF patients at the Clinical of Thoracic and Cardiovascular Surgery of Fann National University Hospital Centre, Dakar, Senegal (control group, healthy individuals) and sequenced. More than half of the *MT-CYB* mutations (58.23%) were heteroplasmic. Transitions (61.67%) were more frequent than transversions (38.33%), and non-synonymous substitutions represented 38.33% of mutations. Unoperated RHD patients harbored frequent *MT-CYB* polymorphisms (7.14 ± 14.70 mutations per sample) and accounted for 72.73% of mutations. Paradoxically, subjects undergoing valvular replacement harbored infrequent polymorphisms (1.39 ± 2.97 mutations per patient) and lacked 36 mutations present in unoperated subjects. A genetic differentiation was observed between these two populations, and the mutations in operated subjects were neutral, while those in unoperated subjects were under positive selection. These results indicate a narrow link (perhaps even causal) between *MT-CYB* mutations and ARF and its complications (i.e., RHDs) and that these mutations are largely deleterious.

## 1. Introduction

Acute rheumatic fever (ARF) is a major cause of cardiac disease and premature death in numerous regions worldwide [1]. ARF and rheumatic heart disease (RHD) have a high prevalence in developing countries in contrast with developed countries where these diseases have largely regressed [2]. ARF results in high morbidity and mortality rates worldwide and its incidence is the highest in Sub-Saharan Africa [3]. ARF incidence in developing countries exceeds 50 per 100,000 children [4]. In Senegal, RHD is the most prominent pediatric cardiovascular pathology [5].

Mutations in numerous genes, particularly those encoding immunity-related factors, are associated with ARF and RHD [6]. Genes encoding pattern recognition receptors belonging to the innate immune system, including ficolin [7], have been investigated. Genes encoding several cytokines including TNF-α or IL-6/-10 were reported to promote or aggravate ARF in studies in Mexico [8,9], Turkey [10], and Egypt [11].

On the other hand, it has been shown that the decrease in oxidative phosphorylation [12] Khatami mainly affects energy-intensive tissues, such as muscles, brain, heart, liver, and kidneys. This mechanism allows the production of ATP by the mitochondrial respiratory chain [13]. *MT-CYB* encodes the cytochrome b protein, which is the only subunit of the respiratory complex III (one of the five complexes of the respiratory chain), encoded by mitochondrial DNA, the others being of nuclear origin [13]. Cytochrome b plays a central role in the production of ATP [12] and as a catalytic subunit binding to the substrate of quinone and facilitating the transmission of electrons to cytochrome c [14]. Many mutations of *MT-CYB*, identified so far, are related to diseases such as stress intolerance, myopathies, cardiomyopathies, and neuropathies. These mutations generally show a deficit of enzymatic activity and a decrease in the number of certain subunits of complex III [13]. Mitochondrial dysfunction is characteristic of heart failure [15].

Thus far, no study on the mitochondrial genome has focused on ARF and RHD, and genetic investigations of these pathologies has not been carried out in Senegal. Hence, we hypothesized that mitochondrial *MT-CYB* mutations influence the occurrence and/or complications in ARF. This study aimed to investigate mutations in *MT-CYB* in ARF and RHD in Senegalese patients. The following were the objectives of our study: (1) To investigate *MT-CYB* polymorphisms in ARF; (2) to evaluate the genetic diversity of *MT-CYB* in ARF; (3) to determine the genetic structure of *MT-CYB* based on populations; (4) to identify the type of *MT-CYB* mutations in ARF.

## 2. Materials and Methods

### 2.1. Study Population

Patients with ARF undergoing follow-up examination at the Clinic of Thoracic and Cardiovascular Surgery of Fann National University Hospital Centre in Dakar, Senegal, were included herein. The study was approved by the ethics and research committee of Cheikh Anta Diop University (reference number: Protocol 0274/2018/CER/UCAD), and patients provided written informed consent prior to their participation in the study in accordance with the tenets of the Declaration of Helsinki. Some of these patients had undergone valvular replacement surgery, while others did not receive surgical intervention. Healthy individuals were recruited as controls.

Patients were divided into three groups: First group, healthy individuals (control group); second group, unoperated ARF patients; third group, operated ARF patients (n = 42 per group). In total, 126 blood samples obtained from each patient were stored in EDTA and labeled as Sg1, Sg2, etc.

### 2.2. Genetic Analysis

#### 2.2.1. DNA Extraction and Amplification and Sequencing of MT-CYB

Genomic DNA was extracted using the DNase Blood Kit (Qiagen, South Korea) in accordance with the manufacturer’s instructions. Polymerase chain reaction (PCR) was carried out to amplify *MT-CYB*, since it is reportedly involved in cardiovascular pathologies [12,15,16,17,18,19]. PCR amplification of *MT-CYB* was carried out at a reaction volume of 50 µL containing 2 µL of concentrated DNA and 48 µL of the PCR mix comprising 29.8 µL of MilliQ water, 5 µL of buffer, 1 µL of supplementary MgCl_2_, 2 µL of dATP, dCTP, dGTP, and dTTP, 5 µL of H15915, 5 µL of L14723, and 0.2 µL of Tap polymerase. L14723 (5’-ACCAATGACATGAAAAATCATGGTT-3’) and H15915 (5’-TCTCCATTTCTGGTTTACAAGAC-3’) were the forward and reverse primers, respectively. The PCR program included the following conditions: 94 °C for 3 min; 40 cycles (94 °C for 45 s; 52 °C for 1 min; 72 °C 1 min for 30 s); 72 °C for 10 min. PCR products were purified and sequenced. Sequencing reactions were performed using an MJ Research PTC-225 Peltier thermocycler with the ABI PRISM kit and electrophoresed in an ABI 3730 XL sequencer.

#### 2.2.2. Molecular Analyses

The chromatograms obtained after sequencing were submitted to the Mutation Surveyor software (https://softgenetics.com) version 5.0 to identify mutations and to determine their nature (homoplasmic or heteroplasmic) and their status (transition or transversion). Sequences of ARF with those of the controls. Mutation Surveyor assigned a score for each mutation, thus indicating the level of confidence regarding the accuracy of the cited base. Only those mutations with a score of ≥20 were retained (the probability that a cited base is false was 0.001; accuracy, 99%).

To determine the appropriate nucleotide position of our mutations in the mitochondrial genome, we performed BLASTn analysis (NCBI; https://ncbi.nlm.nih.gov/) with our raw control sequence. The position of each mutation and the corresponding amino acid was determined using BLASTx 2.8.0 [20], thus facilitating the identification of putative conserved domains [21].

To highlight the potential pathogenicity of non-synonymous mutations, we performed prediction analysis using three different software for transparency and reliability:

- POLYPHEN-2 [22], which yields the following putative results: Probably damaging (p ≤ 5%), potentially damaging (5 < p ≤ 10%), and benign (p > 10%);
- SIFT [23], which assigns a score between zero and one. Amino acid substitutions are predicted to affect protein function when the score is ≤0.05 and to be tolerable when the score is >0.05;
- PROVEAN [24] wherein variants with a score of ≤–2.5 are predicted as deleterious and those with a score of >–2.5 are considered neutral.

Non-synonymous *MT-CYB* mutations have been considered pathogenic if thus stipulated by at least two prediction software; if these mutations are not reported as neutral polymorphisms and if they are present in a conserved domain; heteroplasmy indicates high pathogenicity [25].

For further molecular analysis, we corrected and aligned sequences of ARF patients with those of the controls, using BioEdit version 7.1.9 [26] using the CLUSTALW algorithm [27]. Thereafter, we determined the following parameters underlying the genetic diversity of *MT-CYB* relative to each population, on the basis of which the groups were assessed and differentiated:

- The population size n corresponding to the number of individuals;
- The number of sites N that define the size of sequences;
- The variable sites, invariable sites, non-informative variables, and informative variables, the number of total mutations (Eta), thus elucidating *MT-CYB* polymorphisms in the study population;
- The number of haplotypes and the haplotype diversity (Hd), to analyze the distribution of individuals within a population;
- The nucleotide diversity (Pi) and the average number of nucleotide differences (k) that reflect genetic differences within a population.

These parameters were determined using DNAsp 5.10.01 [28]. Other parameters elucidating the genetic diversity are the following:

- The nucleotide frequencies: General and at each codon position;
- The nature of mutations (transitions and transversions);
- The rate of mutations (R);
- The rate of substitutions: Ks (synonymous) and Kns (non-synonymous).

These parameters were determined using Mega7 software version 7.0.26 [29], and the frequencies of amino acids was determined at the best reading frame (no stop codon).

For analysis using Mega7, it was necessary to determine the model describing the best pattern of substitution; hence, we used the following models:

- The HKY model [30] to control the population;
- The JC model [31] for unoperated ARF patients;
- The JC+G model [31] for operated ARF patients a gamma value of 0.06.

We used the Kimura 2-parameter model [32] to determine the rate of mutations because preferential models were unavailable for this analysis. The Nei-Gojobori modified model was used to estimate substitution rates.

Thereafter, we defined the parameters of genetic diversity among populations, thus highlighting *MT-CYB* polymorphisms in ARF patients. Using the DNAsp software, we estimated the following:

- The rate of nucleotide divergence representing the percentage of nucleotide-level differences in each generation;
- The nucleotide diversity between two populations;
- The average number of nucleotide-level differences at each site between pairwise sequences.

Further, we evaluated genetic differences and structural differences in *MT-CYB* in accordance with the study population. Using Mega7 version 7.0.26 [29], the genetic distances were determined, which facilitated the estimation of the rates of allele replacement among the compared entities, using the JC+G model (with G = 0.36), which was the most suitable model. To estimate genetic distances within populations, we used the K2 model, which is suitable for this analysis, while the JC+G model was not. Furthermore, we estimated the genetic distance between the control population and ARF patients (operated and unoperated). For all analyses of genetic distances, the bootstrap method was used with 1000 replicates and all reading frames were accounted for. Using Arlequin version 3.1 [33], we estimated the genetic differentiation factor (Fst) between populations and performed analysis of molecular variance (AMOVA) to determine the origin of the variants. These analyses were performed considering polymorphic loci only and with 1023 permutations.

The Z-test for selection was performed using Mega7 in accordance with three different alternative hypotheses, the null hypothesis being that H0: dN = dS. We then assessed the neutral selection (H_1_: dN ≠ dS), the positive selection (dN > dS), and the negative selection (dN < dS). We used the Nei-Gojobori modified model [34], which differs from the original method of Nei-Gojobori in that transitional and transversional substitutions were no longer considered to occur at the same frequency. Thus, we calculated the ratio of transitions/transversions with the following formula [30]:

(1) $$\kappa =\frac{\left(\pi t\pi c+\pi a\pi g\right)}{\pi y\pi r}\alpha /\beta$$

where πa, πc, πg, and πt are the nucleotide frequencies (of a, c, g, and t, respectively) estimated by their proportions in all sequences: πy = πt + πc and πr = πa + πg; α and β are the number of transitions and transversions, respectively, observed by considering all sequences in a pairwise manner.

### 2.3. Statistical Analyses

Data normality was assessed using XLSTAT 2018.3.50896 with the Shapiro-Wilk test with a rate of significance level set to 5%. We then performed the Fisher test (for non-normally distributed data) to establish an association between mutations and the state of the disease, always at a threshold of 5%. Mean and standard deviation values for mutations were determined using MS Excel 2010 and compared using R commander (Rcmdr) implemented in RGui version 3.3.4 to assess differences between operated and unoperated ARF patients.

Average amino acid frequencies of the controls and unoperated AFR patients and between the controls and operated ARF patients were compared using the chi-square test in Rstudio version 1.1.447. Since chi-square analysis is only feasible when the number of groups is <5, we used complementary values (100-effective).

## 3. Results

### 3.1. Evaluation of MT-CYB Polymorphisms

#### 3.1.1. Analysis of MT-CYB Mutations

BLASTn analysis revealed 97% identity with *MT-CYB* and 5 gaps. BLASTx analysis revealed 98% identity (without gaps) with the cytochrome b (mitochondrion) [*Homo sapiens*] [35] and [*Homo sapiens subsp. ‘Denisova’*] [36] and 97% identity (without gaps) with cytochrome b (mitochondrion) [*Homo heidelbergensis*] [37] and [*Homo sapiens neanderthalensis*] [38].

Alignment of the chromatograms of ARF patients (operated and unoperated) with those of the controls (Sg97) revealed 165-point mutations with a score of ≥20 or 60 different variants, in the overall study population. Among them, 41.67% corresponded with homoplasmic mutations and 58.23% corresponded with heteroplasmic mutations. Transitions largely surpassed the transversions (respectively 61.67% and 38.33%). Most mutations were present in unoperated ARF patients (72.73%) wherein 7.14 ± 14.70 mutations on average were harbored per individual. Indeed, seven mutations were present at high frequencies in ARF patients (G15849A, T15824C, A15784G, A15362T, A15323C, G15314C, and A15308T). Synonymous (A15362T and A15323C) and missense (G15314C and A15308T) mutations were exclusively present in unoperated ARF patients at frequencies of 67.86%, 67.86%, 42.86%, and 57.14%, respectively. The Fisher test revealed significant results for these mutations, thus indicating their association with ARF. Overall, 36 mutations were absent in the operated ARF patients. Thus, operated ARF patients harbored 1.39 ± 2.97 mutations on average. Differences in the average mutation frequencies were significant (p = 0.031). The frequency for synonymous mutations was 38.33% among the ARF patients. Among these amino acid substitutions, nine were pathogenic according to the predictions of Polyphen-2, SIFT, and PROVEAN. All the pathogenic mutations were absent in the operated patients, except for the missense substitutions K287P and N286P, present in all positions of their corresponding codons and present in only one subject (Sg56). Most mutated sites were detected through MITOMAP (https://www.mitomap.org/MITOMAP); however, the corresponding bases differed from the those in the present sequences (Table 1). 

#### 3.1.2. Variability of Amino Acids

Transcription was carried out based on the second reading frame. Gly and Leu were the most frequent amino acids in the protein. Asn and His were absent in the ARF patients and the controls. Pro and Thr were absent in the controls but present at low frequencies in the ARF patients. However, none of the P-values is significant (Table 2).

#### 3.1.3. Determination of Genetic Diversity of MT-CYB 

The analysis of the genetic diversity of the study population is summarized in Table 3. In the study population, 12, 28, and 54 individuals belonged to the control, unoperated, and operated groups, respectively. The sizes of the sequences were the same for each individual (492 bp). The unoperated group differed from the other two groups and displayed the highest *MT-CYB* polymorphism frequency. The nucleotide frequencies were similar in all groups. Cytosine was the least represented base with a minimal frequency at the third codon position in contrast with thymine and guanine, which were more frequent at this position. Adenine occurred mostly at the first codon position. In position 1, no transversions occurred, and all mutations were transitions (conservation of the mutated base); hence, the mutation rate (R) tended towards infinity. 

#### 3.1.4. Evaluation of the Differentiation and Genetic Structuring of MT-CYB in Accordance with the Study Population

The genetic distance was the largest among unoperated ARF patients (0.011 ± 0.002) and the lowest in the controls (0.003 ± 0.001). Genetic distances were greater between unoperated and operated ARF patients (0.009 ± 0.001) than between the controls and operated ARF patients (0.005 ± 0.001) (Table 4).

Analysis of the genetic differentiation factor (Fst) revealed no genetic differentiation of *MT-CYB* between the controls and patients (p = 0.58559). However, a genetic differentiation of *MT-CYB* between operated and unoperated subjects was observed (p = 0.01466) (Table 5). Nevertheless, AMOVA (Table 6) revealed that more than 98% of genetic variability between unoperated and operated ARF patients is of an intra-population origin.

#### 3.1.5. Evolution of MT-CYB Mutations

As shown in Table 7, the results of the Z selection test among groups were recorded. The non-significant p-values (0.965, 0.483, and 1.000) among operated ARF patients indicate that their mutations follow a neutral evolution. However, among unoperated ARF patients, the p-value, highly significant under the neutrality hypothesis (0.015), highly significant under the hypothesis of positive selection, and not significant under the hypothesis of negative selection, shows that *MT-CYB* mutations in the unoperated patients are under positive selection.

## 4. Discussion

This study attempted to identify *MT-CYB* mutations in Senegalese ARF patients. Since cardiac tissues were not available for molecular analysis, mutations in this region were assessed in peripheral blood. The present results show a high rate of *MT-CYB* polymorphisms in unoperated subjects (72.73% of mutations). Unoperated ARF patients developed a cardiopathy following ARF; however, since they did not undergo valvular replacement, the disease was seemingly induced in them, and *MT-CYB* mutations were detected in the cells involved in the autoimmune response in blood, similar to T and B lymphocytes, and macrophages, since self-reactive T cells migrate from peripheral blood to the heart and proliferate in the valves in response to stimulation by specific cytokines [6].

Two synonymous substitutions (Y206Y and S193S) associated with ARF (according to the Fisher test) were observed at high frequencies (67.86%) and exclusively in the operated subjects. Despite the absence of amino acid substitutions, the corresponding positions (A15362T and A15323C) may serve as potential molecular markers if consistently present in individuals with ARF. Non-synonymous substitutions T190A, F188I, and I185L, frequent in the unoperated group, are also associated with the pathology. These sites were polymorphic in comparison with the control but monomorphic in comparison with the Caucasian reference sequence. These mutations were detected on MITOMAP but matched with different substitutions. The mutation T15301C (I185L) is associated with idiopathic dilated cardiomyopathy [14] as being a synonymous substitution Leu-Leu. Three missense mutations (S344T, L299F, and I298T), predicted to be pathogenic, have been reported in unoperated patients 2 and 1. The substitution of an aliphatic Leu with an aromatic Phe and that of nonpolar Ile with a polar Thr affected protein structure and function. The substitution of Ser with Thr could have been inconsequential, since they are both thiolated/hydroxylated and polar amino acids and the mutation is homoplasmic (the predictions of this mutation are not formal; thus, mutation may be benign). However, these mutations are in a conserved domain, playing a crucial role in cytochrome b function and thus in the activity of complex III. The C-terminal domain contains the binding sites for ubiquinone/ubiquinol [40,41] and is responsible for proton translocation outside the mitochondrial membrane [42]. Furthermore, the L299F mutation has been reported in breast cancer; however, it was a substitution from cytosine to thymine (according to the reference sequence) at position 15641, whereas the mutation detected herein corresponds to the heteroplasmic substitution of guanine by adenine (according to our control sequence). Both mutations were transitions occurring in the same position. Several mutations were present in both unoperated and operated patients, including G15849A and T15824C synonymous substitutions and the A15784G sense mutation. G15849A and T15824C corresponding to substitutions T368I and T360A have been predicted to be non-pathogenic, although they are polar Thr substitutions by non-polar Ile and Ala. These point mutations occur in a conserved domain in mammals, but are outside the C-terminal domain, thus probably accounting for their benign state. T15824C is present in breast cancer as A15824G, and A15784G has been reported in patients with dilated idiopathic cardiomyopathy [16]. Most (60%) of the mutations were absent in operated patients. Mutations present only in the latter were either synonymous or non-pathogenic, except for those present in one patient (Sg56). More than half of the *MT-CYB* mutations were heteroplasmic (58.23%), probably because polymorphonuclear mitochondrial genomes do not contain *MT-CYB* mutations leading to a mixture of wildtype and mutated genomes or heteroplasmy in immune cells. Cells were not isolated for DNA extraction; either of the hypotheses cannot be considered accurate. However, the current speculation that heteroplasmic mutations are more likely to occur in pathogenic mutations rather than in normal polymorphisms [16] is consistent with the present pathogenic mutations.

Amino acid frequencies did not differ significantly upon pairwise group comparisons. However, Pro and Thr were absent in the controls but present in ARF patients at low frequencies, indeed because of the presence of missense mutations.

Differences in the degree of *MT-CYB* polymorphisms between operated and unoperated patients suggest that valvular replacement suppressed gene mutations. This is corroborated by the results of the genetic diversity of the study population, which facilitated the characterization and analysis of differences between unoperated ARF patients and the other two groups in the study population. Similarly, group comparisons in the study population revealed the genetic proximity of operated subjects and the controls (Pi, k, and the genetic distances were low) and those genetic differences between the operated and unoperated patients. These results may have been obtained because surgery eliminates diseased valvular cells and simultaneously the source of the pathology. The proteins of valvular cells similar to those of *Streptococcus pyogenes* [43] are the targets of immune cells. Eradication of the source of the pathology would lead to a correction between the mechanism underlying autoimmunity, and the cells involved in the defense against *Streptococcus* of Group A and autoimmunity would become functional again in the next generation. Selection analyses have indicated that mutations in the operated subjects follow a neutral evolution, while those present in the unoperated patients are under positive selection. These results are concurrent with previous findings; non-synonymous *MT-CYB* mutations tend to consistently occur in unoperated patients. These variants have a deleterious effect on the protein, and an increase in their frequency would further cause protein damage and simultaneously aggravate the disease. Since these mutations generally do not occur in operated patients and those persisting after valvular replacement are neutral (neither beneficial nor deleterious), the involvement of *MT-CYB* mutations in ARF complications can be considered (RHD).

## 5. Conclusions

This study used a population genetics approach to investigate the association of *MT-CYB* mutations with ARF and RHD in Senegalese patients. The present results confirmed our initial hypothesis. Indeed, sixty polymorphic variants of *MT-CYB* were identified herein. Some of these mutations were neutral, while other mutations were pathogenic, as revealed through their effects on cytochrome b structure and function. Furthermore, the absence of more than half of these mutations in patients with valvular replacement and genetic differentiation between the latter and unoperated patients indicates *MT-CYB* polymorphisms, which are closely associated with ARF and RHD. These mutations cause or result from abnormal activation of immune cells against autoantigens. A study combining both immune assessment with a genetics approach would be interesting to clarify why only some individuals infected with group A streptococcus develop an inadequate immune response leading to ARF. A subsequent analysis of the protein would also provide useful insights into the role of cytochrome b in ARF and RHD. In addition, DNA extraction in the same patient before and after surgery could validate our results.

## Figures and Tables

**Table 1:** Characteristics of *MT-CYB* mutations.

Mutations	Rate	p. rCRS	Proportions of Mutations			Status	Nature	p. AA	CD	PolyPhen-2 Prediction	Sift Prediction	Provean Prediction	Conclusion	References
			**S %**	**NO %**	**O%**									
**T69C**	**91**	**15851**	**1.22**	**3.57**	**0.00**	**Homo**	**T**	**I369V**	**Yes**	**Benign 0**	TOL	Neutral	N.P	+ (A > C) (1)
**G71A**	142	15849	9.76	10.71	9.26 *	Homo	T	T368I	Yes	Benign 0	TOL	Neutral	N.P	+ (C > T)
**G81GC**	20	15839	1.22	3.57	0.00	Hetero	T	L365L	Yes					+ (C > T)
**A89AT**	22	15831	1.22	3.57	0.00	Hetero	T	I362I	Yes					
**T94TA**	83	15826	1.22	3.57	0.00	Hetero	T	T360T	Yes					
**T96C**	101	15824	15.85	21.43	12.96 *	Homo	T	T360A	Yes	Benign 0	TOL	Neutral	N.P	+ (A > G)
**T121C**	119	15799	1.22	0.00 *	1.85	Homo	T	Q352Q	Yes					+ (A > G)
**G130A**	103	15790	2.44	0.00	3.70 *	Homo	T	T348T	Yes					+ (C > T)
**A133AC**	21	15787	1.22	0.00 *	1.85	Hetero	T	F347F	Yes					+ (C > T)
**A136G**	86	15784	14.63	10.71 *	16.67 *	Homo	T	P346P	Yes					+ (C > T) (3)
**A136AG**	45	15784	1.22	0.00 *	1.85	Hetero	T	P346P	Yes					+ (C > T) (3)
**A141AT**	25	15779	1.22	3.57	0.00	Hetero	T	Y345Y	Yes					+ (T > C)
**C143G**	150	15777	1.22	3.57	0.00	Homo	T	S344T	Yes	p.D	AFP	Neutral	P	+ (G > C)
**T162C**	146	15758	2.44	0.00	3.70 *	Homo	T	I338V	Yes	Benign	AFP	Neutral	N.P	+ (A > G) (1)
**G171GC**	25	15749	3.66	3.57	3.70	Hetero	T	L335L	Yes					+ (C > T)
**G177GC**	23	15743	1.22	3.57	0.00	Hetero	t	L333L	Yes					+ (C > T)
**C186T**	119	15734	1.22	3.57	0.00	Homo	T	A330T	Yes	Benign	AFP	Neutral	N.P	+ (G > A)
**G187GC**	28	15733	1.22	0.00 *	1.85	Hetero	t	A330A	Yes					+ (C > A)
**A250G**	99	15670	2.44	7.14	0.00 *	Homo	T	H308H	Yes					+ (T > C)
**A254AT**	36	15666	1.22	0.00 *	1.85	Hetero	t	L307L	Yes					
**G256A**	151	15664	1.22	3.57	0.00	Homo	T	I306I	Yes					+ (C > A)
**A257AG**	40	15663	1.22	3.57	0.00	Hetero	T	I306I	Yes					+ (T > C)
**T267TG**	24	15653	1.22	0.00 *	1.85	Hetero	t	M303M	Yes					+ (A > G)
**G279GA**	40	15641	2.44	7.14	0.00 *	Hetero	T	L299F	Yes	P.D	AFP	Neutral	P	+ (C > T)
**A281AG**	**44**	**15639**	**1.22**	**3.57**	**0.00**	**Hetero**	**T**	**I298T**	**Yes**	**P.D**	AFP	Del	P	+ (T > C)
**A287AC**	32	15633	1.22	0.00 *	1.85	Hetero	T	L296L	Yes					
**G288GA**	24	15632	1.22	3.57	0.00	Hetero	T	L296L	Yes					+ (C > T)
**A291AG**	22	15629	1.22	3.57	0.00	Hetero	T	L295L	Yes					+ (T > C)
**G294A**	41	15626	1.22	0.00 *	1.85	Homo	T	L294L	Yes					+ (C > T)
**G304C**	23	15616	1.22	0.00 *	1.85	Homo	T	G290G	Yes					+ (C > T)
**T307C**	48	15613	1.22	0.00 *	1.85	Homo	T	G289G	Yes					+ (A > G)
**T313C**	70	15607	1.22	0.00 *	1.85	Homo	T	K287P	Yes	P.D	AFP	Del	P	(1)(2)(3)
**T314TC**	50	15606	1.22	0.00 *	1.85	Hetero	T	K287P	Yes	P.D	AFP	Del	P	
**T315TC**	50	15605	1.22	0.00 *	1.85	Hetero	T	K287P	Yes	P.D	AFP	Del	P	
**G316GC**	44	15604	1.22	0.00 *	1.85	Hetero	T	N286P	Yes	P.D	TOL	Del	P	+ (C > T)
**T317TC**	45	15603	1.22	0.00 *	1.85	Hetero	T	N286P	Yes	P.D	TOL	Del	P	+ (A > G)
**T318TC**	38	15602	1.22	0.00 *	1.85	Hetero	T	N286P	Yes	P.D	TOL	Del	P	
**A319G**	128	15601	1.22	3.57	0.00	Homo	T	P285P	Yes					+ (T > C)
**A319AG**	58	15601	2.44	7.14	0.00 *	Hetero	T	P285P	Yes					
**G320GC**	22	15600	1.22	0.00 *	1.85	Hetero	T	P285P	Yes					
**G321GC**	106	15599	1.22	0.00 *	1.85	Hetero	T	P285P	Yes					
**A323AG**	24	15597	1.22	3.57	0.00	Hetero	T	V284V	Yes					
**A342G**	23	15578	1.22	3.57	0.00	Hetero	T	Y278Y	Yes					
**G343GC**	35	15577	1.22	3.57	0.00	Hetero	T	A277A	Yes					
**A390AG**	73	15530	1.22	3.57	0.00	Hetero	T	L262L	Yes					+ (T > C) (1)
**C454CT**	31	15466	2.44	7.14	0.00 *	Hetero	T	M240M	Yes					+ (G > A)
**G460GA**	44	15460	1.22	3.57	0.00	Hetero	T	S238L	Yes	Benign	TOL	Neutral	N.P	+ (C > T)
**A466AG**	24	15454	2.44	7.14	0.00 *	Hetero	T	L236L	Yes					+ (T > C)
**G487GA**	24	15433	1.22	3.57	0.00	Hetero	T	A229A	Yes					+ (C > T)
**G534C**	**149**	**15386**	**1.22**	**3.57**	**0.00**	**Homo**	**T**	**H214D**	**Yes**	**Benign**	TOL	Neutral	N.P	+ (C > A)
**G539A**	148	15381	1.22	3.57	0.00	Homo	T	T212I	Yes	Benign	TOL	Neutral	N.P	+ (C > T)
**A558T**	80	15362	23.17	67.86 *	0.00 *	Homo	T	Y206Y	Yes					
**A597C**	128	15323	23.17	67.86 *	0.00 *	Homo	T	S193S	No					+ (G > A)
**G606C**	32	15314	14.63	42.86 *	0.00 *	Homo	T	T190A	No					+ (G > A)
**T609C**	49	15311	3.66	10.71	0.00 *	Homo	T	L189V	No	Benign	TOL	-	N.P	+ (A > G)
**A610AC**	21	15310	1.22	3.57	0.00	Hetero	T	F188I	No					+ (T > C)
**A610GA**	66	15310	1.22	3.57	0.00	Homo	T	F188I	No					+ (T > C)
**A612T**	42	15308	19.51	57.14 *	0.00 *	Homo	T	F188I	No					+ (A > G)
**G616GA**	43	15304	1.22	3.57	0.00	Hetero	T	P186P	No					+ (C > T)
**T619C**	45	15301	3.66	10.71	0.00 *	Homo	T	I185L	No					+ (G > A) (3)

**Rate**: Mutation rates determined via Mutation Surveyor; **p. rCRS**: Position according to the Cambridge reference sequence; **S**: ARF patients; **NO**: Unoperated; **O**: Operated; **p.AA**: Amino acid position according to the protein reference sequence (UniProt accession number: P00156); **CD**: Conserved domain [MT-CYB (52-570) preserved in mammals; Cytochrom_B_C (97-399) preserved in cellular organisms; QcrB (97-564) preserved in cellular organisms; cytochrome_b_C (115-555) containing the redox sites of quinol and the polypeptide binding site, preserved in cellular organisms; MT-CYB6/f-IV (301-435) preserved in cellular organisms]; **Homo**: Homoplasmic mutation; **Hetero**: Heteroplasmic mutation; **T**: Transition; **t**: Transversion; **(1)**: [14] (A > G); **(2)**: [39] (T > C); **(3)**: [16] (A > G); **NP**: Not pathogenic; **P**: Pathogenic; **P.D**: Probably damaging; **p.D**: Potentially damaging; **TOL**: Tolerable; **Del**: Deleterious; *: Significant Fisher; **+**: Listed in MITOMAP, the letters in parentheses represent the referenced substitutions.

**Table 2:** Frequencies of amino acids according to populations.

Amino Acids	Controls	Operated	Non-Operated	P-Value C vs. O	P-Value C vs. NOP	P-Value O vs. NOP
Ala	0.67	0.68	0.69	0.999	0.999	0.999
Cys	4.72	4.68	4.74	0.997	0.999	0.996
Asp	0.00	0.00	0.00	1	1	1
Glu	3.89	3.96	3.96	0.996	0.997	0.999
Phe	2.67	2.70	2.69	0.998	0.999	0.999
Gly	21.40	21.20	21.06	0.987	0.978	0.999
His	0.00	0.00	0.00	1	1	1
Ile	2.67	2.68	2.67	0.999	1	0.992
Lys	2.00	1.93	2.07	0.996	0.996	0.998
Leu	20.68	20.73	20.71	0.997	0.998	1
Met	6.00	6.01	6.00	0.999	0.999	1
Asn	2.67	2.67	2.67	1	1	1
Pro	0.00	0.04	0.07	0.998	0.996	0.998
Gln	1.33	1.34	1.43	1	0.995	0.995
Arg	7.34	7.52	7.46	0.989	0.993	0.996
Ser	3.34	3.36	3.34	0.998	1	0.998
Thr	0.00	0.01	0.05	0.999	0.997	0.999
Val	10.01	9.91	9.89	0.994	0.993	0.999
Trp	8.62	8.57	8.58	0.997	0.998	0.999
Tyr	2.00	2.00	1.93	1	0.996	0.996

C: Controls, O: Operated, NOP: Unoperated.

**Table 3:** Parameters for genetic diversity of the study population.

**Parameters**		Controls				Unoperated				Operated			
**Size of population n**		12				28				54			
**Number of sites N**		492				492				492			
**Non-variables sites**		485				441				450			
Variables sites		7				51				42			
Non-informative variables sites		5				43				25			
Informative variables sites		2				8				17			
Number of total mutations Eta		7				52				52			
Number of haplotypes		7				17				29			
Haplotypic diversity hd		0.833 ± 0.100				0.923 ± 0.037				0.883 ± 0.039			
Nucleotide diversity Pi		0.00314 ± 0.00071				0.00996 ± 0.00336				0.00645 ± 0.00129			
The average number of nucleotide differences k		1.545				4.902				3.173			
Nucleotide frequencies		T	C	A	G	T	C	A	G	T	C	A	G
General		29.5	9.3	25.2	36.0	29.4	9.5	25.3	35.8	29.5	9.5	25.2	35.9
Position 1		21	9.8	27.4	21	9.7	42.1	27.4	21	9.8	42.0	27.3	42.1
Position 2		29	17.1	20.8	32.9	29	17.4	20.9	32.5	29	17.3	20.8	32.6
Position 3		38	1.2	12.7	47.7	38	1.4	12.8	47.5	38	1.3	12.7	47.6
Nature of mutations	Transitions	100				46.92				34.91			
	Transversions	0				53.08				65.09			
Rate of mutations R		∞				0.88				0.54			
Rate of synonymous substitutions Ks		0.002 ± 0.001				0.011 ± 0.003				0.006 ± 0.003			
Rate of non-synonymous substitutions Kns		0.003 ± 0.002				0.007 ± 0.002				0.005 ± 0.002			

**Table 4:** Genetic distances within and among groups.

	Controls	Non-Operated	Operated
Controls	0.003 ± 0.001		
Non-operated	0.007 ± 0.001	**0.011 ± 0.002**	
Operated	0.005 ± 0.001	0.009 ± 0.001	0.007 ± 0.001

In boldface: The highest genetic distances.

**Table 5:** Genetic differentiation factor.

	Controls	Unoperated	Operated
Controls		*0.87195*	*0.38416*
Unoperated	0		***0.01466***
Operated	0.00128	0.01419	

In italics, the p-values and in bold the significant p-value.

**Table 6:** Analysis of molecular variance.

Source of Variation	Percentage Variation
Inter-populations	1.41855
Intra-population	98.58145

**Table 7:** Results of the Z selection test (p-values).

	Neutrality	Positive Selection	Negative Selection
Operated	0.965	0.483	1.000
Non-operated	**0.015**	**0.008**	1.000

In boldface: Significant p-values.
